# Comparative Study on Presentation of Biliary Ascariasis with Dead and Living Worms

**DOI:** 10.4103/1319-3767.65200

**Published:** 2010-07

**Authors:** Shahinul Alam, Golam Mustafa, Salimur Rahman, Shamsul A. Kabir, Harun O. Rashid, Mobin Khan

**Affiliations:** Department of Hepatology, Bangabandhu Sheikh Mujib Medical University, Dhaka, Bangladesh

**Keywords:** Biliary ascariasis, choledocholithiasis, cirrhosis, endoscopy, ERCP

## Abstract

**Background/Aim:**

Ascariasis is a common parasitic infestation in Asia and Latin America. The most serious presentation is biliary and pancreatic ascariasis (BPA). The aim of the present study was to compare the clinical presentation of BPA with dead worms with that with living worms.

**Materials and Methods:**

We included 138 consecutive cases of BPA that occured during the period January 2005 to July 2009. All the patients had endoscopically proven BPA consisting of living or dead worms. Comparison was done by chi-square and independent t tests.

**Results:**

The age (mean ± SD) of the patients was 36.8 ± 16.1 years. Prevalence ratio between male and female patients was 1:5. Ninety eight patients contained living worms and 40 had dead worms. Males were more prone to develop dead worm BPA. The commonest presentation was biliary colic (131; 94.9%); others were acute cholangitis (30; 21.7%), obstructive jaundice (19; 13.8%), choledocholithiasis (20; 14.5%), acute pancreatitis (10; 7.2%), acute cholecystitis (6; 4.3%), liver abscess (2; 1.4%), hepatolithiasis (3; 2.2%), stricture of common bile duct (2; 1.4%), pancreatic abscess (1; 0.7%) and cirrhosis of liver (1; 0.7%). Choledocholithiasis, hepatolithiasis, liver abscess and cirrhosis were associated only with dead worms. We could successfully remove all the worms with endoscopic interventions, but 5 patients required surgical intervention as there were strictures and stones within the biliary tree or Ascaris were in gallbladder. Recurrences of stone and cholangitis occurred only in those with dead worms.

**Conclusion:**

Biliary ascariasis with dead worms is more dangerous than that with living worms. Endoscopic or surgical intervention may be required repeatedly in those with dead worms.

*Ascaris lumbricoides* is a common parasite, and it infects 25% of the world population and 20,000 deaths per year occur due to this.[1,2] The incidence is higher in tropical countries. In the Indian subcontinent, ascariasis is highly endemic in Kashmir (70%), Bangladesh (82%), and central and southwest India (20% to 49%).[3] There are several ways in which intestinal ascariasis can manifest.[3,4] However, the most serious presentation is that of biliary and pancreatic ascariasis (BPA).[5,6] The adult worm occasionally lodges in the common bile duct and produces partial bile duct obstruction. It may be lodged in other parts of biliary canal. Occasionally the worm may die inside the biliary canal.[6] The dead Ascaris may be a nucleus for intrahepatic gallstones.[7] BPA can be associated with living and/ or dead worms. There is scarcity of reports on BPA with dead worms. The present study was designed to compare the clinical presentation and complications of BPA associated with dead worms with those associated with living worms.

## MATERIALS AND METHODS

We documented 138 consecutive cases of BPA that occured during the period January 2005 to July 2009. After initial inclusion of cases with clinical features and sonographic evidence, we finally included the patients who required endoscopic intervention. Informed written consent was taken from every patient. The study was done at the Department of Hepatology, Bangabandhu Sheikh Mujib Medical University, Dhaka, Bangladesh. Ethical clearance was taken from the departmental ethical committee. Exclusion criteria were hemodynamically unstable patients and those with recent myocardial infarction and coagulopathy. Mainstay therapy of biliary ascariasis was conservative, consisting of nil by mouth, analgesics and antibiotics. Endoscopy was done on the day of arrival or on the next day. If a part of the worm was visible outside the ampulla of Vater, it was caught in a Dormia basket and pulled out. Endoscopic retrograde cholangiopancreatography (ERCP) was done under the following circumstances: (1) critically sick with pyogenic cholangitis or unresolving cholecystitis; (2) worms fail to leave the biliary tree within four weeks; (3) worms coexisting with stones; (4) associated liver abscess; (5) biliary ascariasis with intolerable pain and progressively increasing pain. When required, endoscopic sphincterotomy was performed to remove the worms. Six cases did not require sphincterotomy because ampulla was widely open and the basket went inside the common bile duct (CBD) easily. Balloon extraction could not be done in cases of living worms. In cases of dead worms, most of them were torn during extraction that required repeated basket sweeping. There was a follow-up on all patients for 6 to 24 months for any complication or recurrence. Each patient was subsequently given albendazole 400 mg once in a week for two consecutive weeks; and thereafter, every three months.

## RESULTS

A total of 138 patients were included in the study. Their age (mean ±SD) was 36.8 ±16.1 years. Of them, 113 were females and 25 were males. Though females were 5 times more susceptible than males, males were more prone to develop dead- worms ascariasis; out of 25 males, 15 (60.0%) had dead - worms ascariasis. Children were not free from developing biliary ascariasis. Eight (5.7%) of the patients were within 12 years of age, and all of them contained living worms.

**Figure 1:**
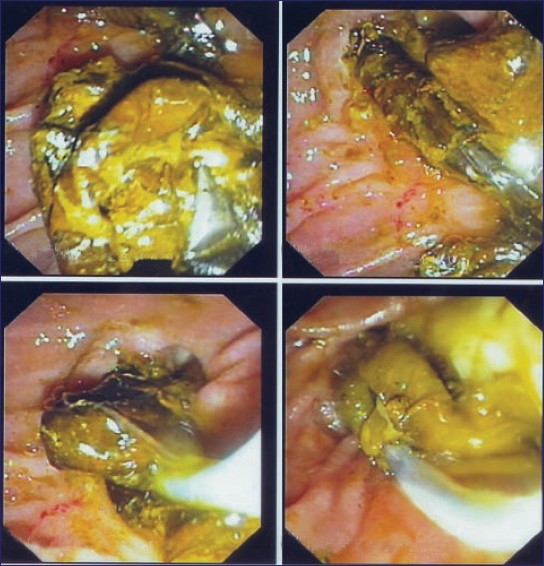
Dead ascariasis and stone in the same patients

Ninety-eight patients had living worms and 40 had dead worms. Commonest presentation was biliary colic (131; 94.9%); others were acute cholangitis (30; 21.7%), obstructive jaundice (19; 13.8%), choledocholithiasis (20; 14.5%), acute pancreatitis (10; 7.2%), acute cholecystitis (6; 4.3%), liver abscess (2; 1.4%), hepatolithiasis (3; 2.2%), stricture of common bile duct (2; 1.4%), pancreatic abscess (1; 0.7%) and cirrhosis of liver (1; 0.7%) [Table 1]. In this study, choledocholithiasis, hepatolithiasis, liver abscess and cirrhosis were associated only with dead worms [Figure 1].

**Table 1:** Presentation of living - worm and dead - worm ascariasis

Character	Living-worm Ascariasis	Dead-worm Ascariasis	*P* value
Age (mean ± SD) years	35.6 ± 17.1	39.7 ± 13.2	0.137
Male/ Female	10 / 88	15 / 25	0.000
Biliary colic	92	39	0.345
Cholangitis	7	23	0.000
Obstructive jaundice	6	13	0.000
Liver abscess	0	2	
Hepatolithiasis	0	2	
Cirrhosis	0	1	
Stricture of CBD	0	2	
Pancreatic abscess	1	0	

**Figure 2:**
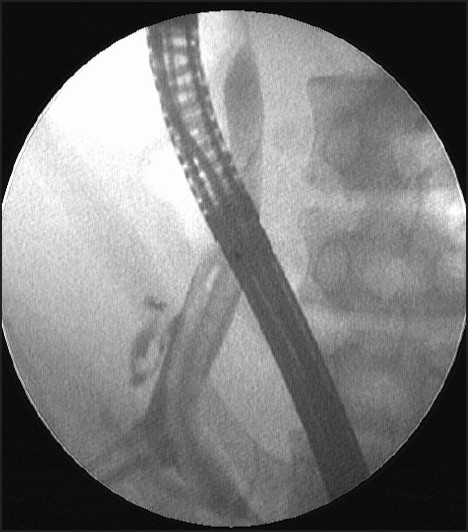
Cholangiogram showing multiple filling defects for *Ascaris lumbricoides*

In all the cases with living worms, we could successfully remove the worms with endoscopic maneuver, but 5 patients had to undergo surgical intervention as there were strictures and stones in 2 cases, and worms were in the gall bladder in 3 cases. Out of 5 patients having worms in gallbladder, worms could be extracted by a basket in 2 patients during ERCP as parts of worms were in the CBD. Single worm was responsible in majority (64.3%) of the cases. Maximum of 21 worms were extracted from 1 (0.7 %) patient. Regarding location of the worms, they were inside the CBD in 75 (54.3%) patients, inside the CBD and common hepatic duct in 24 (17.4%) [Figure 2], inside the CBD and common hepatic duct (CHD) and intrahepatic biliary tree (IHBT) in 14 (10.1%) patients, partially inside and partially outside the CBD in 12 (8.7%) patients, inside gallbladder in 5 (3.6%) patients, inside the pancreatic duct in 5 (3.6%) patients and in the IHBT in 3 (2.2%) patients.

There was a follow-up on all patients for 6 to 24 months after the procedure. One patient had minor bleeding immediately after ERCP, which was controlled spontaneously without requiring blood transfusion. Recurrence of stone and cholangitis occurred in 5 patients who had dead and macerated worms. Recurrence of BPA occurred once in 5 patients and thrice in 2 patients. Two liver abscess cases recovered with medical management after endoscopic removal of worms.

## DISCUSSION

Biliary and pancreatic ascariasis is a common problem in certain parts of the world.[8–11] BPA with dead worms has not been reported previously. This is the first such study on the issue. After entering the bile duct, the Ascaris excrete various types of polypeptides that produce allergic manifestations and cause spasm of the sphincter of Oddi by acting as chemical irritants. The resultant biliary stasis coupled with infected intestinal contents carried by the worms leads to pyogenic cholangitis, cholecystitis and pancreatitis.[12] However, patients infested with worms that have invaded the ampulla of Vater usually present with biliary colic or acute pancreatitis due to blocked CBD or pancreatic duct. These worms migrate through the CBD, pancreatic duct, cystic duct and IHBT, leading to biliary colic, pancreatitis, cholangitis, etc. The presence of a dead worm forms a nidus for stone formation inside the biliary tree. These worms have high glucoronidase activity, which deconjugates bilirubin and forms pigment stones.[13] Biliary ascariasis is predominantly a disease of adult women.[14] These patients usually present with biliary colic (56%), acute cholangitis (25%), acute cholecystitis (13%), acute pancreatitis (6%), and rarely, pancreatic abscess (less than 1%).[15] We have found in our study, in accordance with a previous study,[14,15] that adult females were five times more sufferers than males. Similar presentation was also seen in this series, with biliary colic (94.9%); acute cholangitis (21.7%), obstructive jaundice (13.8%), choledocholithiasis (14.5%), acute pancreatitis (7.2%), acute cholecystitis (4.3%), liver abscess (1.4%), hepatolithiasis (2.2%) and pancreatic abscess (0.7%). But stricture of common bile duct (1.4%) and cirrhosis of liver (0.7%) have not been previously reported.

In this study, one female patient suffered from repeated cholangitis for a period of 11 years. She had hepatosplenomegaly and medium-size esophageal varices; viral markers and metabolic and autoimmune causes were absent. ERCP revealed dead macerated Ascaris with multiple stones. Liver biopsy revealed frank cirrhosis. Here, recurrent cholangitis with obstruction may cause biliary cirrhosis. Does BPA cause cirrhosis of liver? Or could it be associated with oriental cholangiohepatitis? To answer these questions, further evidence is required.

One hundred and thirteen patients (81.9%) in our study were adult females. It is possible that the hormone progesterone in this age group of females leads to relaxation of the smooth muscles of the sphincter of Oddi, allowing easy access of the Ascaris worms into the CBD. Progesterone is a known inhibitor of smooth muscle contraction.[16–20] Though BPA is predominantly a disease of female sex,[5,6,9] male sex is a greater sufferer of advanced disease with dead worms. There is less chance of Ascaris to get entrance inside the CBD of males due to relatively tight sphincter of Oddi, which at the same time makes the CBD a graveyard for Ascaris if ever it enters.

The mainstay therapy of biliary ascariasis is conservative, consisting of nil by mouth, analgesics, antibiotics and repeated de-worming. Majority (80%-90%) of the patients respond to this therapy, and worms return to the duodenum spontaneously. Intervention is required in a small number (<25%) of patients under the following circumstances: (1) those critically sick with pyogenic cholangitis or unresolving cholecystitis; (2) worms fail to leave the biliary tree within four weeks as by that time they are presumed to be dead and need extraction; (3) worms coexisting with stones; (4) associated liver abscess.[21] Endoscopic extraction of worms from the bile duct with or without sphincterotomy, gives immediate relief.[6] In our study, ERCP and papillotomy, as well as basket extraction, were done in 118 patients. Basket extraction only was done in 13 patients; extraction with foreign-body forceps was done in 2 patients; and surgical intervention was required in 5 patients. In another case, surgical intervention was required for pancreatic abscess after endoscopic removal of worms. Re-infection is commonly found in patients who have had previous endoscopic sphincterotomy.[12] We found single recurrence in 3 patients, and recurrence occured thrice in 2 patients. All had previous sphincterotomy.

In conclusion, biliary ascariasis associated with dead worms is more common amongst males than females, but females are greater sufferers of BPA. Stones, abscess, stricture and cirrhosis were associated only with dead worms. Hence biliary ascariasis associated with dead worms is more dangerous than that associated with living worms. It is recommended that prompt measures be taken to treat all cases of biliary ascariasis.
